# The National Medical Guild

**Published:** 1913-09-06

**Authors:** E. Rowland Fothergill

**Affiliations:** Brighton


### THE NATIONAL MEDICAL GUILD.

President: CHAS. BUTTAR, M.A., M.D., B.C.
Gen. Sec.: P. C. RAIMENT, M.R.C.S., L.R.C.P.
Offices: 34 Villiers Street, Strand, London, W.C.

### The Funds of Trade Unions.

*To the Editor of* The Hospital.

Sir,—Would you be good enough to find space for a last communication? In your issue for August 23 you published certain questions framed by myself in order to obtain from the President of the National Medical Guild information as to what were the special powers that it could be reasonably hoped to find in a medical trade union.

These questions were not concisely replied to in that issue, but after a perusal of page 653 of the issue for August 30 your numerous readers will be able to see that these powers are stated to be (1) security of funds, and (2) immunity of officials. Now the latter has been rendered doubtful by a recent judicial pronouncement; and the former is a possibility under the ægis of the British Medical Association without it being necessary to form a medical trade union of that body or for the whole of the medical profession.

With regard to there being any special powers inherent in a *medical* trade union to prevent blacklegs applying for or obtaining appointments below the adopted standard rates of payment, to compel groups of doctors or individual doctors to defer to central collective bargaining, to force employers (individual or collective) of medical labour to pay the standard rates, or to secure the payment by doctors of levies made by their medical trade union; the President of the National Medical Guild practically acknowledges that there are no special powers existing, and that, therefore, a *medical* trade union would have to rely on the honourable conduct of its members; just, in fact, as the British Medical Association has to do at the present time.

This being so, your readers will probably come to the same conclusion as the majority of the members of the medical profession, who, while appreciating the disinterested motives of the National Medical Guild, are satisfied that, as the British Medical Association has held the field for over eighty years, has been acknowledged by the Government of the day, the Insurance Acts Commissioners, and all public authorities throughout the United Kingdom, and has a membership in the United Kingdom of 20,000 out of a possible 30,000, it is far wiser not to organise medical unions, leagues, federations, or guilds, which can at the best only become rivals, but rather to develop the organisation of that Association—scrap-heaping what is effete and obstructive—and at the same time by means of the private, public, and medical schools to emphasise the fundamental fact that honourable conduct amongst medical practitioners is as vital for the interests of the public (individually and collectively) and of medicine as a science as it is for the welfare of any doctor or group of doctors.—I am, etc.,

E. Rowland Fothergill.

Brighton, September 1, 1913.

